# Longitudinal Associations Between Maternal Glucose Levels and Ultrasonographic Fetal Biometrics in a Shanghai Cohort

**DOI:** 10.1001/jamanetworkopen.2022.6407

**Published:** 2022-04-07

**Authors:** Jiao-jiao Zou, Qian Wei, Yu-yang Shi, Ke Wang, Yun-hui Zhang, Hui-jing Shi

**Affiliations:** 1 Key Laboratory of Public Health Safety, Ministry of Education, Department of Maternal, Child and Adolescent Health, School of Public Health, Fudan University, Shanghai, China; 2 Department of Environmental Health, School of Public Health, Fudan University, Shanghai, China

## Abstract

**Importance:**

Evidence on the timing of fetal growth alterations associated with gestational diabetes or on the association of the maternal glycemic trajectory with fetal growth during pregnancy remains lacking.

**Objective:**

To examine the associations between maternal glucose levels and offspring intrauterine growth.

**Design, Setting, and Participants:**

This cohort study used data from 4574 eligible pregnant women and their offspring in the Shanghai Maternal-Child Pairs Cohort collected from April 10, 2016, to April 30, 2018. Group-based trajectory modeling was used to classify fasting plasma glucose levels during pregnancy into 3 glycemic trajectories (trajectory 1, consistently normal glucose levels in all 3 trimesters; trajectory 2, hyperglycemia only in late pregnancy; and trajectory 3, hyperglycemia in all 3 trimesters [ie, consistently high glucose levels]). Statistical analysis was performed from April 25, 2020, to October 1, 2021.

**Exposures:**

Gestational diabetes, which was defined using the results of an oral glucose tolerance test.

**Main Outcomes and Measures:**

Longitudinal fetal biometrics during gestational weeks 11 to 40 and birth outcomes were obtained from medical records. Pregnancy was partitioned into 3 periods (<24, 24-34, and >34 weeks’ gestational age). The differences in offspring growth (log-transformed) and maternal glucose levels were compared using generalized linear mixed models.

**Results:**

A total of 4121 pregnant women had oral glucose tolerance test results (mean [SD] age, 28.8 [4.1] years), 3746 of whom had glycemic trajectory data (mean [SD] age, 28.6 [4.1] years); 983 women (23.8%) had gestational diabetes. Throughout the pregnancy period and compared with the women without gestational diabetes or with women in the trajectory 1 group, the fetal biometrics for the women with gestational diabetes or for those in the trajectory 3 group were significantly higher (except for biparietal diameter), with an estimated increase in fetal weight in the group with gestational diabetes (β = 1.82; 95% CI, 1.03-2.61) and in the trajectory 3 group (β = 1.50; 95% CI, 0.54-2.47; *P* = .002). Fetal biometric alterations among women with gestational diabetes appeared before 24 weeks’ gestational age, with neonatal birth weight significantly higher than in the group without gestational diabetes at 40.4 g (95% CI, 9.8-71.1 g) along with an increased risk of large size for gestational age (odds ratio, 1.36; 95% CI, 1.05-1.75) and macrosomia (odds ratio, 1.47; 95% CI, 1.12-1.94). However, pregnant women in the trajectory 2 group manifested significantly reduced fetal biometrics, and abdominal circumference was significantly augmented after 34 weeks’ gestational age (increase, β = 1.92; 95% CI, 0.87-2.99).

**Conclusions and Relevance:**

In this cohort study, pregnant women who received a diagnosis of gestational diabetes in midpregnancy or had hyperglycemia during all 3 trimesters showed an association with altered fetal growth patterns, including increased estimated fetal weight that appeared before 24 weeks’ gestational age, increased birth weight, and the risk for large size for gestational age and macrosomia.

## Key Points

**Question:**

Are maternal glucose levels associated with the intrauterine growth of offspring?

**Findings:**

In this cohort study of 4574 eligible pregnant women, offspring of mothers with gestational diabetes in midpregnancy or mothers with hyperglycemia in all 3 trimesters were more likely to exhibit altered fetal growth patterns associated with fetal weight and abdominal circumference before 24 weeks of gestation.

**Meaning:**

This study suggests that to ensure normal fetal growth, pregnant women—especially those with high blood glucose levels throughout pregnancy—should not only prevent and control gestational diabetes but also avoid elevated blood glucose levels in late pregnancy.

## Teaser

This cohort study uses data from the Shanghai Maternal-Child Pairs Cohort to examine the associations between maternal glucose levels and offspring intrauterine growth.

## Introduction

An adverse intrauterine environment affects adult metabolism, immune system dynamics, and reproductive function.^1^ Glucose is one of the major components of the maternal intrauterine environment, and according to the fetal programming hypothesis and the developmental origins of health and disease,^2,3,4^ a high glucose intrauterine environment may exert long-term effects by altering an offspring’s genetic imprinting and placental morphologic characteristics and function.^5^ Hyperglycemia during pregnancy can occur once the relationship between insulin levels and glucose metabolism becomes unbalanced^6^; gestational diabetes is a typical manifestation, with a global prevalence of approximately 7% to 26%.^7^ The Hyperglycemia and Adverse Pregnancy Outcomes study^8^ and various case-control studies^9^ have suggested that high glucose levels during any gestational period may increase perinatal complications^10,11,12^; however, few studies have focused on glucose levels during late pregnancy, which is a period that is often neglected by health care systems. Fasting plasma glucose (FPG) levels reflect basal insulin secretion levels, and greater attention to FPG levels may improve maternal outcomes.^9,13^

Gestational diabetes affects more than 10% of pregnancy outcomes,^14^ and studies have shown that metabolic levels of blood glucose may be associated with growth and development during specific periods of pregnancy.^15^ The development and timely implementation of measures to prevent accelerated fetal growth require knowledge of when the trajectory of intrauterine growth begins to deviate. Investigators have reported that accelerated fetal growth starts at 32 weeks among pregnant women with gestational diabetes,^16,17^ whereas some studies have shown that changes in fetal weight associated with gestational diabetes appeared to be present as early as 20 weeks of gestation, as assessed by fetal ultrasonographic scans.^18,19^ Emerging evidence suggests that fetuses in mothers who received a diagnosis of gestational diabetes may have experienced accelerated growth prior to diagnosis.^20^ Moreover, there are significant racial and ethnic differences and significant geographic differences in fetal growth.^21^ The period when the trajectory of fetal growth begins to deviate owing to exposure to maternal glucose remains incompletely understood, and most extant studies have been limited owing to the fact that longitudinal fetal measurements are only rarely available. Thus, the purpose of this study was to investigate the association between maternal blood glucose levels and fetal intrauterine growth at different gestational periods.

## Methods

### Study Population

**Figure 1.**
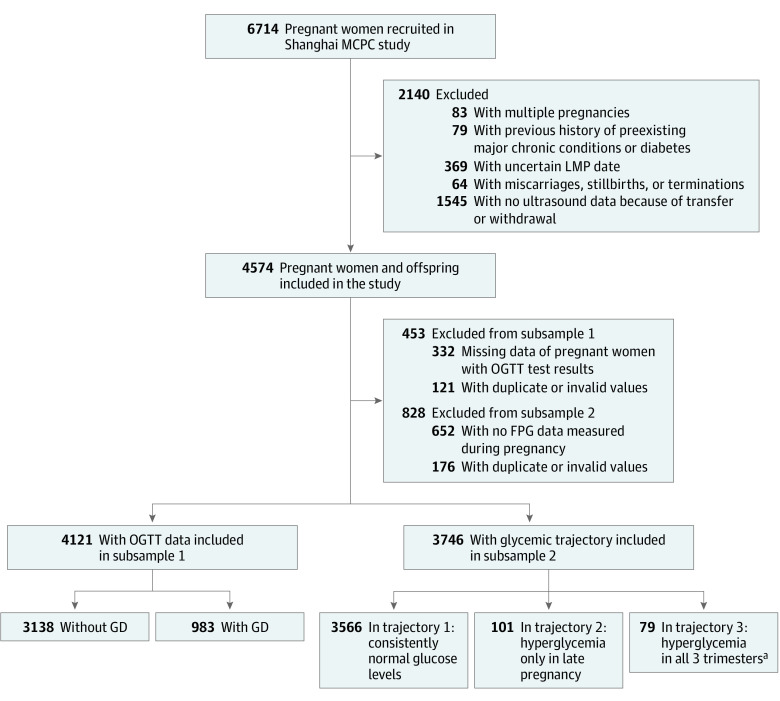
Flow Diagram of Study Participants in the Shanghai Maternal-Child Pairs Cohort (MCPC) Study FPG indicates fasting plasma glucose; GD, gestational diabetes; LMP, last menstrual period; and OGTT, oral glucose tolerance test. ^a^Consistently high glucose levels.

The study was based on the ongoing Shanghai Maternal-Child Pairs Cohort. A total of 6714 pregnant women were recruited at 2 regional maternity hospitals in Shanghai, China, from April 10, 2016, to April 30, 2018. The study included 4574 eligible pregnant women and their offspring; Figure 1 details the participant selection process. The sociodemographic and lifestyle factors of the mother-fetus pairs and other medical information were collected via questionnaires and the medical records of the Maternal and Child Healthcare System. This cohort study followed the Strengthening the Reporting of Observational Studies in Epidemiology (STROBE) reporting guideline. Written informed consent was acquired from all study participants, and this cohort study was approved by the Ethics Committee of the School of Public Health, Fudan University.

### Measurements of Glucose Levels

In routine prenatal examinations, pregnant women were asked to fast overnight for 8 to 10 hours without any food intake (except water), and the hexokinase method (Quailigentglu; Sekisui Chemical Co Ltd) was used to measure FPG levels immediately after blood samples were obtained before breakfast. The FPG levels during pregnancy were assessed during gestational weeks 16, 24, and 33, and analysis was performed by professional medical staff. Pregnant women underwent a 75-g oral glucose tolerance test at approximately 24 weeks’ gestational age that included measurements of FPG as well as plasma glucose levels at 1 hour and 2 hours. Gestational diabetes was defined using International Association of Diabetes and Pregnancy Study Group diagnostic criteria.^22^ Women with abnormal FPG levels or gestational diabetes underwent periodic monitoring and may have been placed on a personalized plan (diet, exercise, and oral hypoglycemic drugs or insulin) to control their blood glucose levels.^23,24^

### Fetal Ultrasonographic Measures and Neonatal Outcomes

Gestational weeks (11-40 weeks’ gestational age) were corrected for the last menstrual period and for the results of the ultrasonographic examination. According to the median distribution of measured gestational weeks, we divided pregnancy into 3 periods with 24 and 34 weeks’ gestational age as the nodes (ie, <24, 24-34, and >34 weeks’ gestational age). Data on fetal biometrics (abdominal circumference [AC], head circumference [HC], humerus length, femur length [FL], and biparietal diameter [BPD]) (eTable 1 in the Supplement) and birth anthropometrics (length and weight) were retrieved from the medical records. Fetal biometrics had multiple ultrasonographic values (median, 4 [IQR, 1-6]) that were measured (using Voluson E8; GE Healthcare) by trained sonographers.^25^ The estimated fetal weight (EFW) was calculated using the formula of Hadlock et al^26^ based on AC, FL, and HC. Small size for gestational age was defined as a birth weight in the 10th percentile or lower, and large size for gestational age (LGA) was defined as a birth weight in the 90th percentile or higher according to birth weight reference percentiles for Chinese citizens.^27^ Macrosomia implied growth beyond an absolute birth weight that is historically 4000 g, whereas a birth weight lower than 2500 g was defined as low birth weight.

### Covariates

Maternal characteristics, such as prepregnancy weight, height, parity, maternal age, delivery mode, gestational weeks at delivery, last menstrual period, and weight before delivery, were extracted from the medical records. Prepregnancy body mass index (BMI; calculated as weight in kilograms divided by height in meters squared) was categorized into 3 groups (<18.5, 18.5-23.9, and ≥24.0).^28^ Mothers’ gestational weight gain was calculated as the difference between prepregnancy weight and weight before delivery.^29^ Data on educational level, total family income, parental smoking or drinking status, history of diabetes, and father’s age, height, and weight were collected by questionnaire. Maternal sleep quality, level of physical activity, anxiety, and depression during pregnancy were evaluated using professional scales or questionnaires described previously.^30^ Maternal energy intake in late pregnancy was calculated by food-exchange units using the information collected with the semiquantitative Food Frequency Questionnaire.^31^ Pregnancy complications were defined as preexisting conditions among pregnant women.^32^

### Statistical Analysis

Statistical analysis was performed from April 25, 2020, to October 1, 2021. We first applied the group-based trajectory model to identify subgroups of women based on their FPG levels measured during the first, second, and third trimesters.^33^ The number of FPG subgroups and the functional model of each group were identified by the following principles: (1) a bayesian information criterion (requiring data as close to 0 as possible), (2) the threshold of the mean posterior probability of membership (reaching ≥0.7 for each FPG trajectory subgroup), and (3) a reasonable distribution of the participants across subgroups. After fitting with a quadratic term and linear term, the result of the group-based trajectory model identified 3 subgroups based on 3774 pregnant women who differed from each other in their glycemic fluctuation during 3 trimesters (entropy = 0.84; bayesian information criterion = −3719.21). Entropy is required to be greater than 0.7, indicating that the model was well fit. The 3 FPG subgroups were as follows: trajectory 1, consistently normal glucose levels (72.0-90.0 mg/dL [to convert to millimoles per liter, multiply by 0.0555]) in all 3 trimesters (95.2% [3566 of 3746]; mean posterior probability, 0.95); trajectory 2, hyperglycemia (glucose level >91.8 mg/dL) only in late pregnancy (2.7% [101 of 3746]; mean posterior probability, 0.94); and trajectory 3, hyperglycemia (>91.8 mg/dL) in all 3 trimesters (ie, consistently high glucose levels) (2.1% [79 of 3746]; mean posterior probability, 0.80). Moreover, we found fewer than 10 women whose blood glucose level changed from high to low during pregnancy, and therefore we removed them from the study analysis. Second, the fetal measures were log transformed to stabilize variances across gestational age and approximate normal distributions. To better explain the regression model results after log transformation of the outcome variables, we converted all regression coefficients (β) and 95% CIs to percentage change using the formula (*e^β^* − 1) × 100%. We exploited generalized linear mixed models to compare the differences in the longitudinal associations of fetal biometric measurements among maternal glucose level groups in overall pregnancy (fixed effects: adjusted for covariates related to the pregnant women and their husbands, gestational weeks at delivery, sex, and birth information [no interactions were included]) and during the 3 pregnancy periods (fixed effects: additionally adjusted for measured gestational weeks and the square of the measured gestational weeks). The random-effects model incorporates a within-individual repeated-measures error and between-individual random effects. The generalized linear regression model was used to examine the associations between maternal glucose level groups and birth outcomes. Sensitivity analyses were performed using alternative Hadlock formulas based on BPD, AC, and FL (EFW, formula 2) and based on BPD, AC, HC, and FL (EFW, formula 3). We also repeated the analyses with sex stratification as another sensitivity index. Missing data for some of the covariates were imputed using multiple imputation (20 imputed data sets). Significance tests were 2-tailed, and *P* < .05 was considered to be statistically significant. We performed all analyses using Stata, version 16.0 (StataCorp LLC) and R, version 4.0.5 (R Group for Statistical Computing).

## Results

A total of 4121 pregnant women had oral glucose tolerance test results (mean [SD] age, 28.8 [4.1] years), 3746 of whom had glycemic trajectory data (mean [SD] age, 28.6 [4.1] years) (Table 1). Women with gestational diabetes (n = 983 [23.8%]) tended to be older at delivery (mean [SD] age, 30.0 [4.3] vs 28.4 [4.0] years), gained less weight during pregnancy (mean [SD], 13.8 [5.4] vs 15.1 [5.1] kg), and exhibited a higher prepregnancy BMI relative to the women without gestational diabetes. In these indices, the trajectory 3 and trajectory 1 groups were similar to the groups with or without gestational diabetes. The incidence of LGA was 9.9% (365 of 3678), the incidence of small size for gestational age was 5.5% (201 of 3678), the incidence of macrosomia was 6.5% (239 of 3678), and the incidence of low birth weight was 2.7% (100 of 3678).

**Table 1.** Characteristics of Participants

Variable	Participants, No. (%)					*P* value
	Subsample 1 with OGTT data		Subsample 2 with glycemic trajectory group^a^			
	No gestational diabetes (n = 3138)	Gestational diabetes (n = 983)	Trajectory 1 (n = 3566)	Trajectory 2 (n = 101)	Trajectory 3 (n = 79)	
Parents’ characteristics						
Maternal age, mean (SD), y	28.4 (4.0)	30.0 (4.3)	28.6 (4.1)	28.0 (4.4)	29.9 (4.8)	.02
Gestational weight gain, mean (SD), kg	15.1 (5.1)	13.8 (5.4)	15.0 (5.2)	14.5 (5.2)	13.7 (5.8)	.05
FPG level in early pregnancy, mean (SD), mg/dL	81.5 (7.6)	85.5 (9.4)	81.7 (7.0)	81.7 (6.8)	106.4 (13.1)	<.001
FPG level in middle pregnancy, mean (SD), mg/dL	78.8 (5.6)	86.6 (11.0)	79.9 (6.3)	80.1 (6.5)	105.3 (11.9)	<.001
Plasma glucose level, mean (SD), mg/dL						
1 h	128.3 (25.0)	169.4 (34.7)	135.4 (30.8)	133.7 (29.5)	182.0 (45.2)	<.001
2 h	113.6 (19.4)	140.0 (31.3)	118.4 (24.3)	117.4 (24.3)	142.9 (35.8)	<.001
FPG level in late pregnancy, mean (SD), mg/dL	77.4 (15.5)	83.5 (14.8)	74.3 (9.2)	117.7 (17.5)	99.4 (9.4)	<.001
Prepregnancy BMI						
<18.5	580 (18.5)	121 (12.3)	603 (16.9)	13 (12.9)	13 (16.5)	.002
≥24.0	442 (14.1)	224 (22.4)	556 (15.6)	31 (30.7)	13 (16.5)	
Educational level >12 y	1306 (41.6)	429 (43.6)	1441 (40.4)	24 (23.8)	43 (54.4)	.26
Total family income ≤¥200 000^b^	2319 (73.9)	711 (72.3)	2640 (74.0)	65 (64.4)	78 (98.7)	.50
Primiparous	1837 (58.5)	505 (51.4)	2024 (56.8)	46 (45.5)	55 (69.6)	.87
Depression in late pregnancy	385 (12.3)	115 (11.7)	418 (11.7)	12 (11.9)	7 (8.9)	.21
Anxiety in late pregnancy	380 (12.1)	104 (10.6)	410 (11.5)	5 (5.0)	11 (13.9)	.36
Energy intake in late pregnancy, mean (SD), kcal	2258.0 (1214.8)	2020.5 (1077.2)	2199.1 (1193.6)	2121.9 (1161.4)	2152.0 (1618.4)	.80
Complications	427 (13.6)	114 (11.6)	468 (13.1)	12 (11.9)	10 (12.7)	.56
Good sleep quality in late pregnancy	1180 (37.6)	425 (43.2)	1404 (39.4)	32 (31.7)	53 (67.1)	.20
Low PA level in late pregnancy	1271 (40.5)	375 (38.2)	1453 (40.8)	36 (35.6)	39 (49.4)	.22
Father’s age, mean (SD), y	29.5 (4.5)	30.93 (5.0)	29.7 (4.7)	28.9 (4.7)	31.3 (5.7)	.001
Father’s BMI, mean (SD)	23.7 (3.3)	24.0 (3.5)	23.7 (3.3)	23.8 (3.5)	24.2 (3.4)	.32
Offspring characteristics						
Male sex	1602 (51.1)	496 (50.5)	1838 (51.5)	37 (36.6)	50 (63.3)	.66
Gestational age at delivery, mean (SD), wk	39.2 (1.3)	38.9 (1.3)	39.1 (1.3)	38.7 (2.1)	38.9 (1.3)	.003
Cesarean delivery	1589 (50.6)	536 (54.5)	1844 (51.7)	21 (20.8)	59 (74.7)	<.001

Abbreviations: BMI, body mass index (calculated as weight in kilograms divided by height in meters squared); FPG, fasting plasma glucose; OGTT, oral glucose tolerance test; PA, physical activity.

SI conversion factor: To convert glucose to millimoles per liter, multiply by 0.0555.

^a^ Trajectory 1, consistently normal glucose levels in all 3 trimesters; trajectory 2, hyperglycemia only in late pregnancy; and trajectory 3, hyperglycemia in all 3 trimesters (ie, consistently high glucose levels).

^b^ The current exchange rate of $1 to ¥6.32 was used.

Before 24 weeks’ gestational age, the mean (SD) EFW (348.2 g [95% CI, 340.5-356.0 g] vs 346.0 g [95% CI, 342.5-349.5 g]), AC (155.6 mm [95% CI, 154.2-157.0 mm] vs 154.6 mm [95% CI, 154.0-155.3 mm]), HC (172.4 mm [95% CI, 170.9-173.9 mm] vs 171.6 mm [95% CI, 170.9-172.3 mm]), FL (31.1 mm [95% CI, 30.7-31.5 mm] vs 31.1 mm [95% CI, 30.9-31.3 mm]), and BPD (48.4 mm [95% CI, 48.0-48.9 mm] vs 48.0 mm [95% CI, 47.8-48.3 mm]) were significantly greater in the group with gestational diabetes than in the group without gestational diabetes (Table 2). These comparisons all remained significant after correction for multiple comparisons. Specifically, the mean (SD) fetal EFW was larger in the trajectory 3 group than in the trajectory 1 group before 24 weeks’ gestational age (451.8 g [95% CI, 428.0-476.8 g] vs 362.8 g [95% CI, 358.8-366.9 g]) and after 34 weeks’ gestational age (2889.0 g [95% CI, 2874.9-2903.2 g] vs 2814.8 g [95% CI, 2727.9-2904.5 g]).

**Table 2.** Geometric Mean of Fetal Growth Characteristics

Variable	Geometric mean (95% CI)		*P* value^a^	Geometric mean (95% CI)			*P* value^a^
	Subsample 1 with OGTT data			Subsample 2 with glycemic trajectory group			
	No gestational diabetes	Gestational diabetes		Trajectory 1	Trajectory 2	Trajectory 3	
<24 Weeks’ gestational age							
Total No.^b^	9894	2009		7437	207	154	
EFW, g	346.0 (342.5-349.5)	348.2 (340.5-356.0)	.002^c^	362.8 (358.8-366.9)	365.7 (341.3-391.9)	451.8 (428.0-476.8)	.92
AC, mm	154.6 (154.0-155.3)	155.6 (154.2-157.0)	.004^c^	158.5 (157.8-159.2)	159.0 (154.5-163.7)	169.3 (164.8-174.0)	.71
HC, mm	171.6 (170.9-172.3)	172.4 (170.9-173.9)	.002^c^	176.2 (175.4-177.0)	174.8 (170.1-179.6)	190.0 (185.8-194.3)	.78
HL, mm	30.1 (29.9-30.2)	30.0 (29.7-30.4)	.002^c^	30.9 (30.7-31.1)	31.0 (29.9-32.0)	34.7 (33.9-35.6)	.98
FL, mm	31.1 (30.9-31.3)	31.1 (30.7-31.5)	.002^c^	32.2 (32.0-32.4)	32.0 (30.9-33.2)	36.4 (35.5-37.4)	.98
BPD, mm	48.0 (47.8-48.3)	48.4 (48.0-48.9)	.002^c^	49.4 (49.2-49.6)	49.5 (48.3-50.8)	52.9 (51.8-53.9)	.71
24-34 Weeks’ gestational age							
Total No.^b^	5243	1094		3673	105	51	
EFW, g	1437.4 (1421.8-1453.1)	1410.8 (1374.6-1447.9)	.97	1411.6 (1390.5-1433.0)	1370.9 (978.7-1920.3)	1402.6 (1273.8-1544.4)	.22
AC, mm	258.3 (257.2-259.3)	256.0 (253.6-258.4)	.85	256.4 (255.0-257.9)	249.6 (221.3-281.5)	253.8 (245.9-262.0)	.20
HC, mm	270.9 (270.0-271.8)	269.0 (267.0-271.1)	.23	269.7 (268.5-271.0)	265.3 (242.6-290.1)	262.9 (255.7-270.3)	.20
HL, mm	49.8 (49.7-50.0)	49.6 (49.3-50.0)	.12	50.0 (49.8-50.2)	51.7 (50.2-53.2)	48.9 (47.8-50.1)	.23
FL, mm	56.0 (55.8-56.2)	55.6 (55.2-56.1)	.12	56.3 (56.0-56.5)	58.4 (56.4-60.5)	55.0 (53.6-56.5)	.22
BPD, mm	78.1 (77.8-78.3)	77.6 (77.0-78.1)	.14	78.3 (78.0-78.6)	80.3 (77.8-82.9)	76.8 (74.9-78.7)	.20
>34 Weeks’ gestational age							
Total No.^b^	8216	1626		6235	163	117	
EFW, g	2871.1 (2849.0-2893.2)	2880.3 (2868.9-2891.6)	.23	2814.8 (2727.9-2904.5)	2682.2 (2633.3-2732.0)	2889.0 (2874.9-2903.2)	.61
AC, mm	327.8 (327.3-328.3)	327.9 (326.9-329.0)	.35	327.9 (327.2-328.6)	320.2 (316.4-324.0)	325.5 (321.4-329.7)	.80
HC, mm	321.1 (320.8-321.5)	319.9 (319.3-320.6)	.006^c^	321.5 (321.1-321.9)	302.7 (285.7-320.7)	316.7 (314.1-319.3)	.02^d^
HL, mm	60.5 (60.5-60.6)	60.4 (60.3-60.6)	.29	60.7 (60.6-60.8)	61.0 (60.3-61.7)	60.3 (59.7-60.9)	.43
FL, mm	69.6 (69.5-69.7)	69.4 (69.3-69.6)	.29	69.8 (69.7-70.0)	70.4 (69.7-71.1)	69.3 (68.7-70.0)	.43
BPD, mm	92.6 (92.5-92.7)	92.5 (92.3-92.7)	.07	92.7 (92.6-92.8)	91.8 (91.0-92.7)	92.0 (91.4-92.7)	.43

Abbreviations: AC, abdominal circumference; BPD, biparietal diameter; EFW, estimated fetal weight; FL, femur length; HC, head circumference; HL, humerus length; OGTT, oral glucose tolerance test.

^a^ A false discovery rate less than 0.05 and *P* < .05 were considered significant.

^b^ Total number of each measured fetal biometric marker.

^c^ *P* < .01.

^d^ *P* < .05.

**Figure 2.**
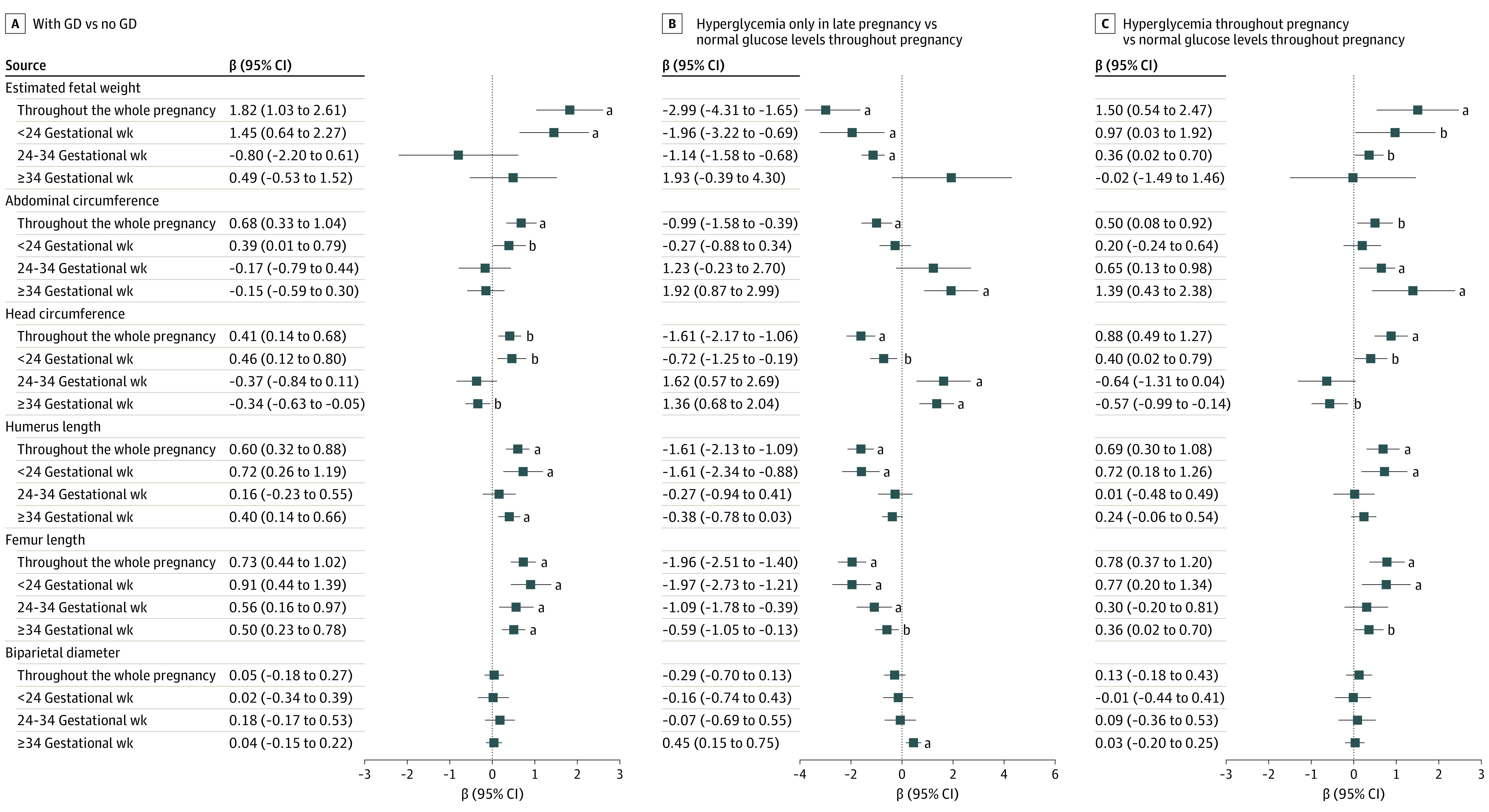
Comparisons of Fetal Biometric Measurements by Maternal Glucose Level Groups During Pregnancy A, Mothers with gestational diabetes (GD) vs those with no GD. B, Mothers with hyperglycemia only in late pregnancy vs those with normal glucose levels in all 3 trimesters. C, Mothers with hyperglycemia in all 3 trimesters vs those with normal glucose levels in all 3 trimesters. Fetal biometric measurements are brought into the model after log-transformed conversion. Measurements throughout the whole pregnancy indicate adjustment for sex, educational level, income, maternal age, prepregnancy body mass index category, maternal depression, anxiety, physical activity level, sleep quality, energy intake in late pregnancy, history of diabetes, gestational weight gain, parity, gestational weeks at delivery, father’s body mass index, and father’s smoking or drinking. The 3 pregnancy periods are additionally adjusted for measured gestational weeks and the square of the measured gestational weeks instead of gestational weeks at delivery. ^a^*P* < .01. ^b^*P* < .05.

Associations between groups with different maternal glucose levels and different fetal biometrics based on a longitudinal analysis across pregnancy periods are depicted in Figure 2. Offspring in the group with gestational diabetes exhibited higher EFW (β = 1.82; 95% CI, 1.03-2.61) and larger AC (β = 0.68; 95% CI, 0.33-1.04) than offspring in the group without gestational diabetes. The results were similar in the trajectory 3 group in that fetal EFW (β = 1.50; 95% CI, 0.54-2.47) and AC (β = 0.50; 95% CI, 0.08-0.92) were higher than in the trajectory 1 group. However, biometric indices (except for biparietal diameter) were reduced in the trajectory 2 group relative to the trajectory 1 group.

Further stratification of the 3 pregnancy periods revealed differences in fetal biometric measurements between the group with and the group without gestational diabetes. The fetal biometrics of pregnant women with gestational diabetes exhibited an identical increasing trend between the period before 24 weeks’ gestational age and the entire pregnancy. Offspring of mothers with gestational diabetes showed a higher mean EFW (β = 1.45; 95% CI 0.64-2.27) and AC (β = 0.39; 95% CI, 0.01-0.79) than offspring of mothers without gestational diabetes before 24 weeks’ gestational age. Comparisons of fetal biometrics between the trajectory 3 group and the trajectory 1 group revealed that the trajectory 3 group was associated with a mean EFW increase before 24 weeks’ gestational age (β = 0.97; 95% CI, 0.03-1.92) and at 24 to 34 weeks’ gestational age (β = 0.36; 95% CI, 0.02-0.70), as well as an increase in the mean fetal AC after the fetus reached term at 24 to 34 weeks’ gestational age (β = 0.65; 95% CI, 0.13-0.98) and after 34 weeks’ gestational age (β = 1.39, 95% CI, 0.43-2.38). Moreover, HC, humerus length, and FL all increased before 24 weeks’ gestational age, similar to the index changes over the entirety of pregnancy. The results of the comparison between the trajectory 2 group and the trajectory 1 group were virtually opposite to those of the trajectory 3 and trajectory 1 groups during the entirety of pregnancy and during the 3 pregnancy periods. The increase in AC among women in the trajectory 2 group began after 34 weeks’ gestational age (β = 1.92; 95% CI, 0.87-2.99), and the mean FLs were reduced in all 3 periods. The sensitivity analyses did not materially alter the primary results (eTable 2 and eFigure in the Supplement).

Neonates exposed to gestational diabetes were 40.4 g (95% CI, 9.8-71.1 g) heavier at birth and showed an increased risk of LGA (odds ratio, 1.36; 95% CI, 1.05-1.75) and macrosomia (odds ratio, 1.47; 95% CI, 1.12-1.94) compared with those born to mothers without gestational diabetes (Table 3). Compared with the trajectory 1 group, the trajectory 3 group showed an increase in birth weight by 92.9 g (95% CI, 12.9-172.9 g) and had an increased risk of LGA (odds ratio, 2.24; 95% CI, 1.30-3.89) and macrosomia (odds ratio, 2.20; 95% CI, 1.20-4.03) in their offspring.

**Table 3.** Associations of Glucose-Level Groups With Offspring Birth Outcomes^a^

Variable	Gestational diabetes vs no gestational diabetes	Trajectory 2 vs trajectory 1^b^	Trajectory 3 vs trajectory 1^b^
Outcome as continuous variable, β (95% CI)			
Birth weight in grams	40.43 (9.79 to 71.07)	–36.15 (–124.09 to 51.79)	92.88 (12.88 to 172.89)
Birth length in centimeters	0.06 (–0.01 to 0.13)	–0.23 (–0.44 to –0.02)	0.13 (–0.07 to 0.32)
Outcome as categorical variable, OR (95% CI)			
SGA	0.85 (0.58 to 1.24)	0.91 (0.32 to 2.55)	0.43 (0.10 to 1.78)
LGA	1.36 (1.05 to 1.75)	0.59 (0.21 to 1.65)	2.24 (1.30 to 3.89)
Macrosomia	1.47 (1.12 to 1.94)	1.10 (0.50 to 2.41)	2.20 (1.20 to 4.03)
LBW	0.81 (0.44 to 1.51)	1.32 (0.35 to 5.02)	0.76 (0.15 to 3.71)

Abbreviations: LBW, low birth weight; LGA, large size for gestational age; OR, odds ratio; SGA, small size for gestational age.

^a^ Adjusted for sex, educational level, income, maternal age, maternal smoking or drinking, depression, anxiety, physical activity level, sleep quality, energy intake in late pregnancy, history of diabetes, prepregnancy body mass index category, gestational weight gain, parity, gestational age, delivery mode, and father’s body mass index.

^b^ Trajectory 1, consistently normal glucose levels in all 3 trimesters; trajectory 2, hyperglycemia only in late pregnancy; and trajectory 3, hyperglycemia in all 3 trimesters (ie, consistently high glucose levels).

## Discussion

In this prospective cohort study, maternal glucose levels during pregnancy were associated with fetal growth and neonatal birth outcomes. Maternal gestational diabetes and hyperglycemia in all 3 trimesters produced a mean increase in fetal biometric indices (EFW, HC, humerus length, and FL) and in the risk for LGA and macrosomia. Furthermore, mothers with hyperglycemia only in late pregnancy had augmented fetal AC after 34 weeks’ gestational age. Collectively, our findings indicated an association between maternal glucose levels and intrauterine fetal growth, with the association beginning before the diagnosis of gestational diabetes.

The developing fetus is particularly sensitive to the intrauterine environment, and gestational hyperglycemia can promote fetal growth, with changes starting to emerge as the fetus reaches term before 24 weeks’ gestational age. Measurements of fetal growth with gestational diabetes exposure before or after 24 weeks’ gestational age, however, show inconsistent results. Results from Li and colleagues^34^ were similar to ours, with all fetal biometric growth percentiles significantly higher among women with gestational diabetes than women with normal blood glucose levels at a mean of 20.6 weeks’ gestational age. Macaulay et al^35^ demonstrated an increase in offspring growth at 16 weeks’ gestation owing to gestational diabetes exposure, especially with respect to AC. In contrast, 3 studies revealed that fetal measurements, such as AC and EFW, were smaller or showed no differences before 24 weeks’ gestational age among women with gestational diabetes but were then elevated after this period.^20,36,37^ In contradistinction, no significant differences in EFW or AC between the gestational diabetes and control groups were observed in the second and third trimesters in a previous study.^36^ These results differed from ours and may be due to their small sample size, insufficient number of fetal longitudinal measurements, and disparities in races and ethnicities and in the diagnosis of gestational diabetes.^37,38^ In addition, pregnant women may receive a systematic clinical intervention after the diagnosis of gestational diabetes^39^; this intervention may bring about transitory alterations in all fetal biometrics as characterized by greater intrauterine growth in the gestational diabetes group before 24 weeks’ gestational age, after which the difference disappears.

The glycemic level during pregnancy and the intrauterine development of offspring are both continuous processes. Existing studies focused on the association of blood glucose level in the second trimester with intrauterine development, without fully considering blood glucose level in the first trimester. In this study, we demonstrated that if the mothers’ glycemic levels were normal in the first and second trimesters of pregnancy and increased in late pregnancy, the fetuses tended to be smaller and yet showed an increased AC. If the glycemic level continued to increase from early pregnancy, the AC of the offspring also exhibited a continuous increase from midpregnancy, and this increase in AC may portend future metabolic risks.^40^ The blood glucose level in early pregnancy is also important. A suboptimal glucose metabolism in early pregnancy may also be associated with fetal fat development and bone growth.^20,37,41^ One study has suggested that the glycemic level continues to be high in early pregnancy, with intrauterine growth in the first and second trimesters decreasing^42^; however, the midpregnancy blood glucose level was ignored, and the measurement index that was used instead was the nonfasting plasma glucose level, which may have caused the divergence between the results of that study and ours. Although offspring growth has been shown to be closely associated with maternal glycemic level, it is also associated with other factors, such as the placental capacity for glucose transport and placental glucose metabolism.^43^

Birth outcome is also associated with hyperglycemia during pregnancy. In our study, offspring birth size and the risk of LGA and macrosomia among women with gestational diabetes and women with hyperglycemia had increased, which was of clinical significance. Evaluations of an African cohort uncovered no difference in neonatal birth weight between women with and women without gestational diabetes.^35^ Genotype is responsible for 15% of birth weight, and some posit that fetal growth can be abnormal before gestational diabetes is diagnosed. Evidence has shown that poor blood glucose control in early pregnancy presents an increased risk for macrosomia and developmental delays in offspring.^41,42,44^ Although some pregnant women have undergone clinical interventions after receiving a diagnosis of gestational diabetes, the association of higher glucose levels during pregnancy with birth outcomes persists, and the incidence of LGA remains high.^45^ Another study revealed that, compared with a diagnosis of gestational diabetes without treatment, the risk of adverse birth outcomes was significantly reduced with treatment for gestational diabetes.^39,46^ The Pedersen hypothesis suggests that the net effect of birth weight gain is attributable to the growth-promoting effects of insulin and glucose, and thus blood glucose control in late pregnancy is also essential.^47^ Attention to the blood glucose level throughout pregnancy is therefore critical from the perspectives of cause and prevention.

### Strengths and Limitations

Our study has several strengths. It used data from a population-based prospective cohort that included maternal blood glucose levels and fetal longitudinal growth data throughout pregnancy. Owing to the propensity of glucose levels to change during the day and to be sensitive to the intake of carbohydrates, we used FPG levels to avoid this potential data bias. The confounding variables of the mother’s lifestyle during pregnancy and the father’s demographic information were also controlled to ensure the stability of the results as much as possible. We focused on maternal glucose levels in all 3 trimesters, and fetal growth measurements were taken before and after receiving a diagnosis of gestational diabetes, which had clinical implications for the prevention of adverse fetal growth patterns.

This study also had some limitations. The intrauterine growth profiles that we observed may not have reflected actual growth patterns because the pregnant women with gestational diabetes in our study had undergone partial treatments. Furthermore, fetal ultrasonographic measurements were inevitably affected by the inability to eliminate measurement errors, although we controlled for the main confounders. We used only FPG instead of other blood glucose indicators to reflect pregnancy blood glucose levels, although studies have shown that FPG level is associated with offspring growth.^48,49^ The lack of an FPG subgroup from high to low during pregnancy was another limitation. Future studies are required to validate our findings with more detailed maternal glucose measurements obtained from women prior to becoming pregnant.

## Conclusions

Through multiple longitudinal measurements of intrauterine growth, our study concluded that gestational diabetes and hyperglycemia during all 3 trimesters were associated with accelerated fetal growth that manifested prior to the diagnosis of gestational diabetes as well as with elevated risks of LGA and macrosomia. The study provided further evidence for early onset of fetal overgrowth associated with intrauterine hyperglycemia. It may be necessary to rethink when to screen for gestational diabetes in order to take precautions in early pregnancy to prevent excessive fetal growth. Monitoring the blood glucose level throughout pregnancy was a crucial component in assessing offspring growth patterns, especially for those pregnant women who did not receive a diagnosis of gestational diabetes but who had hyperglycemia in late pregnancy because their offspring had significantly increased AC after 34 weeks’ gestational age.

## Notes

Supplement. **eTable 1.** Descriptive Statistics of the Repeat Ultrasound Fetal Biometry Measurements in the Cohort **eTable 2.** Sensitivity Analysis of Fetal Biometry Measurements in Alternative Hadlock Formulas (β (95% *CI*)) **eFigure.** Comparisons of Fetal Biometric Measurements by Maternal Glucose Level Groups During Pregnancy
