# Supplementary material for: Longitudinal Associations Between Maternal Glucose Levels and Ultrasonographic Fetal Biometrics in a Shanghai Cohort
Source: JAMA Netw Open. 2022 Apr 7;5(4):e226407. doi: 10.1001/jamanetworkopen.2022.6407 (PMC8990351; doi:10.1001/jamanetworkopen.2022.6407)
Supplement: Supplement. — eTable 1. Descriptive Statistics of the Repeat Ultrasound Fetal Biometry Measurements in the Cohort eTable 2. Sensitivity Analysis of Fetal Biometry Measurements in Alternative Hadlock Formulas (β (95% CI)) eFigure. Comparisons of Fetal Biometric Measurements by Maternal Glucose Level Groups During Pregnancy [file jamanetwopen-e226407-s001.pdf]

Supplementary Online Content

Zou J, Wei Q, Shi Y, Wang K, Zhang Y, Shi H. Longitudinal associations between maternal glucose levels and ultrasonographic fetal biometrics in a Shanghai cohort. *JAMA Netw Open.* 2022;5(4):e226407. doi:10.1001/jamanetworkopen.2022.6407

eTable 1. Descriptive Statistics of the Repeat Ultrasound Fetal Biometry Measurements in the Cohort
eTable 2. Sensitivity Analysis of Fetal Biometry Measurements in Alternative Hadlock Formulas (β (95% *CI*))
eFigure. Comparisons of Fetal Biometric Measurements by Maternal Glucose Level Groups During Pregnancy

This supplementary material has been provided by the authors to give readers additional information about their work.

The total no. of sample measurements for EFW, AC, HC, HL, FL, and BDP in this study were 21523, 21663, 21696, 25697, 25735, and 26000, respectively. (eTable 1)

eTable 1. Descriptive Statistics of the Repeat Ultrasound Fetal Biometry Measurements in the Cohort

Measurements	N with at least three measurements	Total no. of measurements	Median (INQ) no. of measurements per individual	Minimum and maximum no. of measurements per individual
Estimated Fetal Weight	18787	21523	4(1~6)	1,14
Abdominal Circumference	18903	21663	4(1~6)	1,14
Head Circumference	18914	21696	4(1~6)	1,14
Humerus Length	23635	25697	4(3~6)	1,14
Femur Length	23692	25735	4(3~6)	1,14
Biparietal Diameter	24020	26000	4(3~6)	1,16

Sensitivity analysis results:

Results from different EFW calculation formulas were in keeping with the effects of glucose levels in our present EFW findings (eTable 2). The hyperglycemic group (GDM and the Trajectory 3 group) exhibited a fetal growth of girls identical to that of both sexes during the whole of pregnancy, while the results from boys showed that EFW, HL, and FL increased during pregnancy, with the effects appearing before week 24 of gestation. The effect of glucose on AC in the Traj2 group was more pronounced in females, and the trend was consistent with our present findings (eFigure 1).

© 2022 Zou J et al. *JAMA Network Open.*

eTable 2. Sensitivity Analysis of Fetal Biometry Measurements in Alternative Hadlock Formulas (β (95% *CI*))

	**With GDM vs. no GDM**	**Traj2 vs. Traj1**	**Traj3 vs. Traj1**
**EFW2 (Total no. of Measurements=21811)**			
Throughout the whole pregnancy	0.96 (0.23, 1.69)	-3.00 (-4.34, -1.64)	1.54 (0.56, 2.52)
<24 gestational weeks	1.22 (0.37, 2.07)	-2.12 (-3.40, -0.82)	1.06 (0.11, 2.03)
24~34 gestational weeks	-1.08 (-2.47, 0.34)	5.44 (2.08, 8.91)	1.99 (-0.30, 4.34)
≥34 gestational weeks	0.45 (-0.55, 1.46)	-3.05 (-5.07, -1.00)	-0.02 (-1.47, 1.44)
**EFW3 (Total no. of Measurements=21787)**			
Throughout the whole pregnancy	0.87 (0.14, 1.61)	-2.85 (-4.21, -1.48)	1.44 (0.46, 2.43)
<24 gestational weeks	1.12 (0.26, 1.98)	-2.03 (-3.33, -0.72)	0.97 (0.01, 1.95)
24~34 gestational weeks	0.46 (-0.55, 1.47)	5.41 (2.01, 8.93)	2.77 (0.46, 5.14)
≥34 gestational weeks	0.31 (-0.68, 1.31)	-2.95 (-5.00, -0.85)	-0.41 (-1.86, 1.05)

Trajectory 1, consistently normal glucose levels in all 3 trimesters; Trajectory 2, hyperglycemia only in late pregnancy; and Trajectory 3, hyperglycemia in all 3 trimesters [ie. consistently high glucose levels].

Estimated fetal weight 2 based on BDP, AC and FL; Estimated fetal weight 3 based on BDP, AC, HC and FL.

Fetal biometry measurements are brought into the model after log-transformed conversion. * p<0.05, ** p<0.01.

Throughout the whole pregnancy adjusted for sex, education, income, maternal age, pre-pregnancy BMI category, maternal depression, anxiety, PA level, sleep quality, energy intake in late pregnancy, history of diabetes, gestational weight gain, parity, gestational weeks at delivery, father's BMI, father smoking or drinking.

The three trimesters additionally adjusted measured gestational weeks and square of measured gestational weeks instead of gestational weeks at delivery.

© 2022 Zou J et al. *JAMA Network Open.*

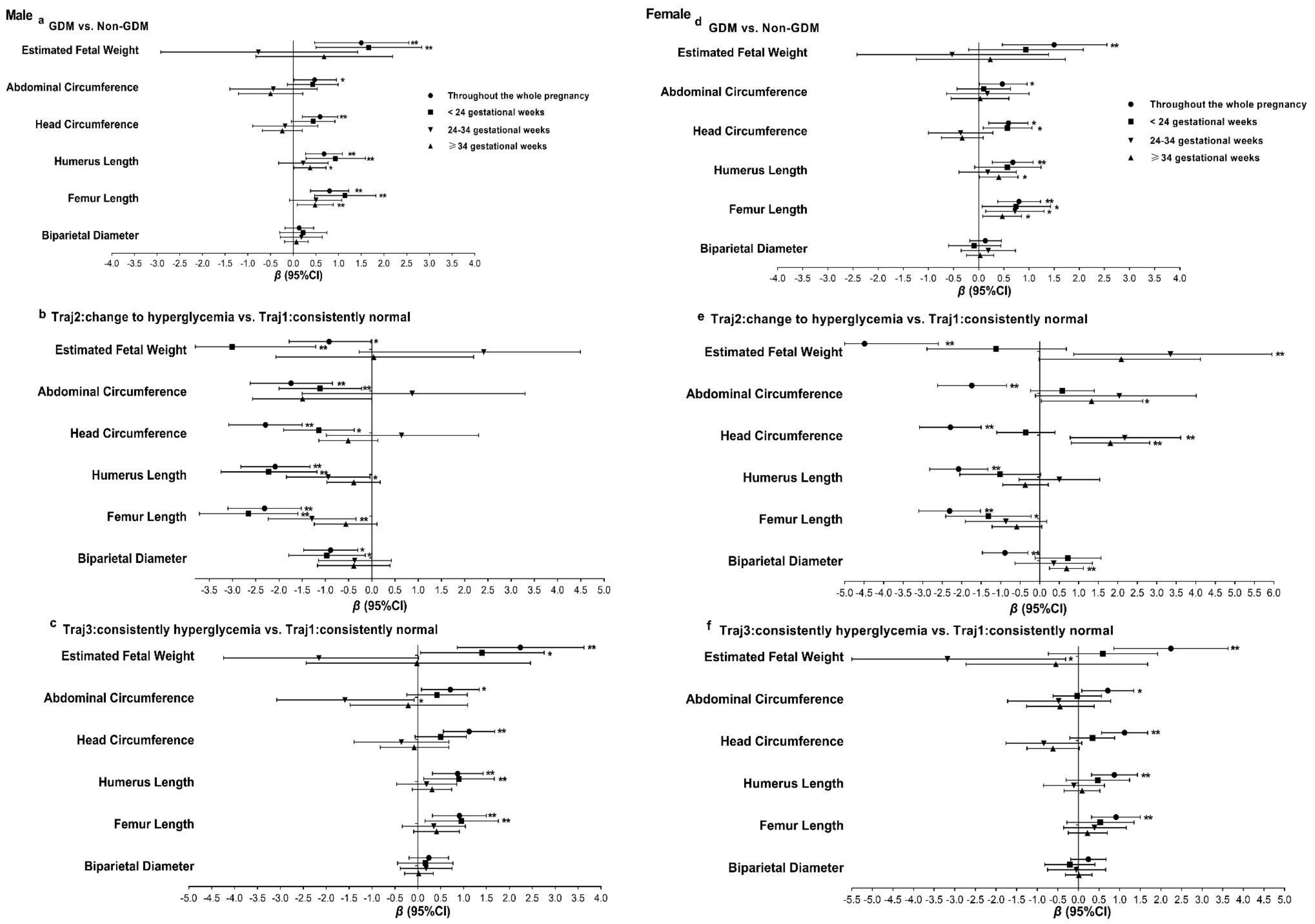

© 2022 Zou J et al. *JAMA Network Open.*

eFigure. Comparisons of Fetal Biometric Measurements by Maternal Glucose Level Groups During Pregnancy (sex stratification as another sensitivity analysis)

Male: a,b,c ; Female: d,e,f

Fetal biometry measurements are brought into the model after log-transformed conversion. * p<0.05, ** p<0.01.

Circles adjusted for education, income, maternal age, pre-pregnancy BMI category, maternal depression, anxiety, PA level, sleep quality, energy intake in late pregnancy, history of diabetes, gestational weight gain, parity, gestational weeks at delivery, father's BMI, father smoking or drinking.

The three periods additionally adjusted measured gestational weeks and square of measured gestational weeks instead of gestational weeks at delivery.

© 2022 Zou J et al. *JAMA Network Open.*
